# On the Origin of Tetraploid Vernal Grasses (*Anthoxanthum*) in Europe

**DOI:** 10.3390/genes12070966

**Published:** 2021-06-24

**Authors:** Zuzana Chumová, Terezie Mandáková, Pavel Trávníček

**Affiliations:** 1 Czech Academy of Sciences, Institute of Botany, CZ-242 53 Průhonice, Czech Republic; pavel.travnicek@ibot.cas.cz; 2 Department of Botany, Faculty of Science, Charles University, Benátská 2, CZ-128 00 Prague, Czech Republic; 3 CEITEC, Masaryk University, CZ-625 00 Brno, Czech Republic; Terezie.Mandakova@ceitec.muni.cz; 4 Department of Experimental Biology, Faculty of Science, Masaryk University, CZ-625 00 Brno, Czech Republic

**Keywords:** FISH, flow cytometry, GBSSI, genome size, GISH, Poaceae, polyploidy

## Abstract

Polyploidy has played a crucial role in the evolution of many plant taxa, namely in higher latitudinal zones. Surprisingly, after several decades of an intensive research on polyploids, there are still common polyploid species whose evolutionary history is virtually unknown. Here, we addressed the origin of sweet vernal grass (*Anthoxanthum odoratum*) using flow cytometry, DNA sequencing, and in situ hybridization-based cytogenetic techniques. An allotetraploid and polytopic origin of the species has been verified. The chromosome study reveals an extensive variation between the European populations. In contrast, an autopolyploid origin of the rarer tetraploid vernal grass species, *A. alpinum*, has been corroborated. Diploid *A. alpinum* played an essential role in the polyploidization of both European tetraploids studied.

## 1. Introduction

*Anthoxanthum* L., the vernal grass, is a genus of the family Poaceae, comprising annual and perennial taxa that are often self-incompatible [1] and show large diversity in ploidy and morphology (e.g., [2,3]). Despite being a relatively small genus standing out of main research attention within the Poaceae, it offers a great opportunity to study different modes of polyploidization and their consequences for the further evolution of the entire genus.

The genus *Anthoxanthum* is distributed mainly in temperate and arctic-alpine regions of northern Eurasia, the Mediterranean, Macaronesia, and East Africa. The delimitation of several species within the genus is controversial (e.g., [2,4,5,6,7]), but the most traditional taxonomic concepts [5,6,8] recognize seven *Anthoxanthum* species in Europe, three annuals (*A*. *aristatum* Bois., *A*. *gracile* Biv., and *A*. *ovatum* Lag.) and four perennials (comprising *A*. *alpinum* A. Löve et D. Löve, *A*. *maderense* Teppner, *A*. *odoratum* L., and *A*. *amarum* Brot.). Following Pimentel et al. [9], where two sections of *Anthoxanthum* are recognized (sect. *Ataxia* and sect. *Anthoxanthum*), two additional species (besides all European) are included in the sect. *Anthoxanthum* in Africa: the East African-endemic *A. nivale* K.Schum. [10,11] and an endemic species of Ethiopian mountains *A. aethiopicum* Hedberg [12]. In addition to species appearing in the abovementioned taxonomic literature, one additional taxon (called “Mediterranean diploid” hereafter) was recognized [3,13,14]. Most species are diploids, but polyploidy has been found in some populations of *A. alpinum* (tetraploids), all populations of *A. odoratum* (tetraploids), *A. amarum* (high polyploids), as well as *A. aethiopicum* (tetraploid) and *A. nivale* (tetraploids, octoploids, and dodecaploids) [3,10,11,12,13,15,16,17,18].

Sweet vernal grass (*A. odoratum*) represents the most widespread *Anthoxanthum* species that grows throughout Europe and has been introduced in North and South America, Australia, Tasmania, and New Zealand. It is also known from KwaZulu-Natal and Eastern Cape in South Africa [19] and is introduced as a fodder grass to some countries.

There has been a long-standing debate as to whether 4*x A*. *odoratum* originated via auto- or allo-polyploidization. An autotetraploid origin of *A. odoratum* has been suggested much earlier than the second type based on the meiotic investigation and high frequency of quadrivalents [20,21,22]. Additionally, Hedberg [23] supported this hypothesis based on the occurrence of triploid individuals within diploid populations. However, after the discovery of another diploid karyotype (“Mediterranean diploid”, [14]) and based on the crossing experiments [24,25], allo-polyploidization turned out to be a more likely hypothesis. Assuming the additivity of genome sizes, Chumová et al. have also found the autopolyploid hypothesis unlikely in the previous study [3].

Mainly on the basis of genetic and phenotypic evidence Borrill [1] has concluded that *A. odoratum* could have arisen from the hybridization of *A. alpinum* and *A. ovatum* or their closely related ancestors. To verify Borrills’ conclusions, Jones [14] did a chromosome study on *A. odoratum*. His results were that “neither karyotype nor meiotic pairing contains any support for the supposition that *A. odoratum* is an autotetraploid and unless it be assumed that the species has undergone an extreme degree of chromosome differentiation, it must be concluded that it has a hybrid origin”. As a possible scenario based on the karyotypes of the diploids *A. aristatum*, *A. ovatum,* and an unnamed species from Crete (“Mediterranean diploid”), he suggested that *A. odoratum* could have evolved from such crosses as *A. alpinum* × *A. ovatum*, *A. alpinum* × “Mediterranean diploid” or *A. ovatum* × “Mediterranean diploid”. Additionally, Teppner [26] and Hedberg [25,27] proposed a hypothesis of doubling after hybridization between two different diploids (*A. alpinum* and the “Mediterranean diploid”). Our previous data based on the genome sizes [3] have also shown the diploid *A. alpinum* (5.52 pg) to be one parent of *A. odoratum* and the “Mediterranean diploid” (7.42 pg) the second one, if we accept the additive model of genome size values. Nevertheless, our data cannot exclude the participation of *A. aristatum/ovatum* or *A. maderense* in the genesis of the tetraploid as the second parent of *A. odoratum*.

To confirm the allopolyploid hypothesis and to suggest possible scenarios of the origin of *A. odoratum*, we have built on our previous studies [3,13] and involved analyses of nuclear and plastid DNA, and fluorescence and genomic in situ hybridization (FISH and GISH, respectively) that have been used in many studies to resolve the origins of polyploids before (e.g., [28,29,30,31]).

Furthermore, we involved tetraploid *A. alpinum* in the analyses. This cytotype is distributed in the Southern Jura, on the western part of the Alps and in the Massif Central, where it replaces the diploid form [26,32]. Its autopolyploid origin has been suggested several times [3,24,26,32,33,34] and, therefore, seemed to provide a good comparative material in this case.

In fact, the tetraploid accessions of *Anthoxanthum* have been analyzed in the frame given by comprehensive study on diploids [13] supplemented with nuclear low-copy gene phylogeny and GISH analyses. Such an approach, combining data on diploids with new data for tetraploids, allows us to reveal the relationships between tetraploids and diploids, determine the most likely origin of tetraploids and elucidate the consequences of polyploid evolution in *Anthoxanthum*.

## 2. Materials and Methods

### 2.1. Plant Material

**Figure 1:**
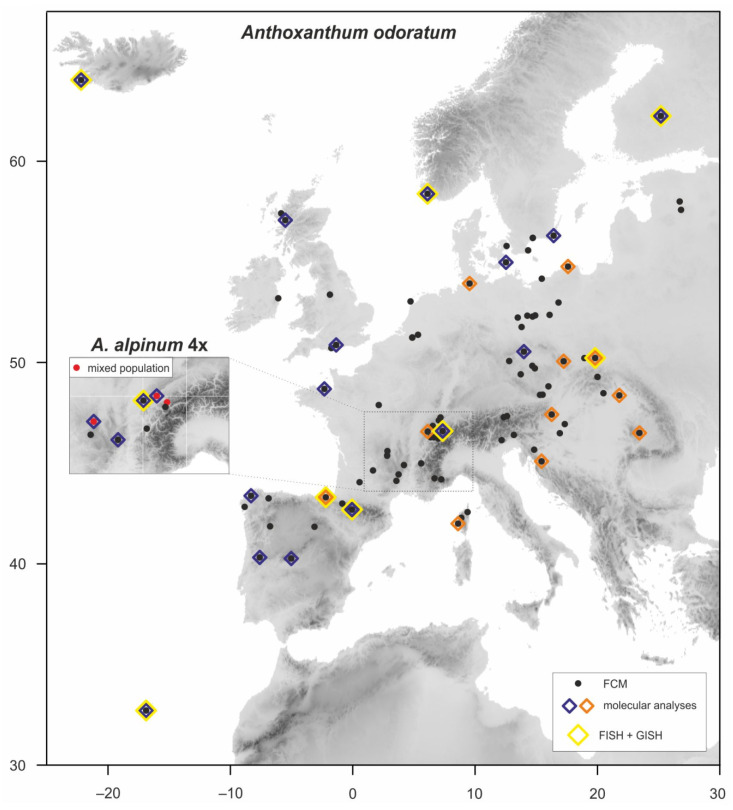
Map showing the locations of 103 populations of *Anthoxanthum odoratum* and 8 populations of tetraploid *A. alpinum* (in the inset cutout) included in the study. A supplementary color legend highlights the use of selected individuals in various analyses.

The *Anthoxanthum odoratum* and tetraploid *A. alpinum* material was collected in 2006–2019 throughout the species’ range in Europe (Figure 1, Supplementary Table S1). Ploidy level of all plants was determined by flow cytometry to separate them from the diploid morphologically similar species, and both cytotypes were validated by chromosome counts. In addition to the new collections, all populations of these two species from our previous study [3] were included here and 39 diploid populations (each population represented by one specimen) from Chumová et al. [13] were used in the molecular and genomic analyses.

### 2.2. Flow Cytometry

Holoploid and monoploid genome sizes [35] were estimated by means of propidium iodide FCM. For each plant, one young, intact leaf, approximately 1 cm in length, was chopped along with an appropriate amount of an internal reference standard using a new razor blade in a Petri dish containing 0.5 mL of ice-cold Otto I buffer (0.1 M citric acid, 0.5% Tween 20) [36,37]. The resulting suspension was filtered through a 42-µm nylon mesh and incubated at room temperature for at least 5 min. After incubation, the suspension was stained by using 1 mL of Otto II buffer (0.4 M Na_2_HPO_4_·12 H_2_O) supplemented with the intercalating fluorescent dye propidium iodide, RNAase IIA (both at the final concentrations of 50 µg·mL^−1^) and β-mercaptoethanol (2 µL·mL^−1^). The samples were stained for 5 min at room temperature and analyzed using a Partec CyFlow cytometer (Partec GmbH, Münster, Germany) equipped with a 532 nm diode-pumped solid-state laser Cobolt Samba (Cobolt AB, Solna, Sweden) as the source of excitation light. Fluorescence intensity of 5000 particles was recorded, and the data were analyzed using Partec FloMax Software version 2.4d. *Pisum sativum* ‘Ctirad’ (2C = 8.76 pg; [38]) served as the reference standard. In total, 275 *Anthoxanthum* plants (242 individuals of *A. odoratum* and additional 33 individuals of tetraploid *A. alpinum*) were subjected to FCM analysis, and their DNA tetraploid level (*sensu* Suda et al. [39]) was inferred from their estimated DNA C-values, using karyologically verified plants as reference points.

To test the association between genome sizes and geographic locations (latitude, longitude and altitude) of sampled populations the Pearson’s correlation in R 3.6.3 [40] was used.

### 2.3. Molecular Data Collection

All molecular analyses were performed on 39 selected diploid individuals used in previous study [13], 28 (cpDNA)/23 (GBSSI) selected individuals of *A. odoratum* and 4 individuals of *A. alpinum* 4*x* (Figure 1, Supplementary Table S1). We analyzed two plastid regions (*trnL-trnF* and *rpl32-trnL*) and the Granule-bound starch synthase (GBSSI or waxy) gene of nuclear DNA (nrDNA). Total genomic DNA was extracted from 0.5 g of dried leaf tissue using the DNeasy Plant Mini Kit (Qiagen, Valencia, CA, USA). The plastid region *trnL-trnF* was amplified and sequenced using primers *c* and *f* (*trnL* intron plus *trnL-trnF* intergenic spacer, hereafter *trnL-F*; [41]). The PCR conditions for the *trnL-F* region were as follows: 1 min of denaturation at 94 °C, followed by 34 cycles at 94 °C for 50 s, 50 s at 52.5 °C, 90 s at 72 °C, and a final extension of 10 min at 72 °C. The plastid region *rpl32-trnL* was amplified and sequenced using primers *trnL^(UAG)^* and *rpL32-F* under the PCR conditions following [42]. Finally, the GBSSI gene was amplified and sequenced using primers *F-for* and *M-bac* under the PCR conditions following [43]: 1 min of denaturation at 94 °C, followed by 35 cycles at 94 °C for 50 s, 50 s at 65 °C, 2 min at 72 °C and a final extension of 20 min at 72 °C. Due to the occurrence of within-individual polymorphisms in some of the directly sequenced PCR products of the GBSSI gene, PCR products for GBSSI were cloned using the pGEM-T Easy Vector System (Promega, Madison, WI, USA) following the manufacturer’s instructions but downscaled to half-volume reactions. Details of the procedure were as described by Záveská et al. [44]. A total of 16 colonies from each individual were used as templates for PCR and sequencing. PCRs of all loci were done with MyTaq polymerase (Bioline, London, UK) or AmpliTaq Gold DNA Polymerase (Applied Biosystems, Foster City, CA, USA), following the manufacturer’s instructions, but with the annealing temperatures mentioned above. PCR products were purified with the Jetquick PCR Purification Spin Kit (Genomed, Warsaw, Poland) and directly sequenced at Macrogen Inc. (Seoul, South Korea) or at the DNA sequencing laboratory of the Biological Section of Charles University, with the original PCR primer sets in both directions.

### 2.4. Molecular Data Analyses (cpDNA and nrDNA)

The two datasets of sequences, i.e., the concatenated chloroplast DNA (cpDNA) dataset and the GBSSI dataset, were aligned independently using MAFFT 7 [45], and then the alignments were improved manually in BioEdit v.7.0.0. [46]. Polymorphisms found in a particular alignment in only one sequence were considered as polymerase errors and corrected [47]. GBSSI sequences from the cloned PCR products were inspected for presence of PCR or in vivo recombinants according to Záveská et al. [44], and a maximum of four GBSSI alleles per tetraploid were kept for further analyses.

Phylogenetic trees were reconstructed using Bayesian analyses (BA) accomplished with MrBayes v.3.1.2. [48]. The datasets were tested for the best substitution model using jModelTest v.0.1.1 [49] with default settings. We chose GTR + G + I model (i.e., MrBayes setting to nst = 6 rates = invgamma) according to the Bayesian information criterion (BIC; [50,51]) for both of them. Two parallel runs with four chains each were used, sampling every 100th tree for 8 million generations for each dataset. The first 10% of samples (8000 trees) were discarded as burn-in, and the remaining 72,000 trees per run were summarized. Nodes with posterior probability (PP) values of 0.95 and above were regarded as significant and those with PP values below 0.95 regarded as non-significant.

### 2.5. Chromosome Preparations

In total, eight populations of *A. odoratum* and one population of tetraploid *A. alpinum* were chosen for FISH and GISH analysis (see Table S1). Mitotic chromosome spreads were prepared from root tips as described by [3]. Briefly, root tips were harvested from germinating seeds or cultivated plants, pre-treated with ice-cold water for 16 h, fixed in ethanol/acetic acid (3:1) fixative for 24 h at 4 °C and stored at −20 °C until further use. Selected root tips were rinsed in distilled water (twice for 5 min) and citrate buffer (10 mM sodium citrate, pH 4.8; twice for 5 min), and digested in 0.3% cellulase, cytohelicase and pectolyase (all Sigma–Aldrich) in citrate buffer at 37 °C for 3 h. After digestion, individual root tips were dissected on a microscope slide in 20 μL acetic acid and spread on the slide placed on a metal hot plate (50 °C) for c. 30 s. Then, the preparation was fixed in freshly prepared ethanol/acetic acid (3:1) fixative by dropping the fixative around the drop of acetic acid and into it. The preparation was dried using a hair dryer and staged using a phase contrast microscope. Chromosome preparations were treated with 100 μg·mL^−1^. RNase in 2 × sodium saline citrate (SSC; 20 × SSC: 3 M sodium chloride, 300 mM trisodium citrate, pH 7.0) for 60 min and with 0.1 mg·mL^−1^ pepsin in 0.01 M HCl at 37 °C for 5 min; then postfixed in 4% formaldehyde in 2 × SSC for 10 min, washed in 2 × SSC twice for 5 min, and dehydrated in an ethanol series (70%, 90%, and 100%, 2 min each).

### 2.6. DNA Probes

The BAC clone T15P10 (AF167571) of *Arabidopsis thaliana* bearing 45S rRNA gene repeats was used for in situ localization of nucleolar organizer regions (NOR), and the *A. thaliana* clone pCT 4.2 (M65137), corresponding to a 500 bp 5S rRNA repeat, was used for localization of 5S rDNA loci. For GISH, total genomic DNA (gDNA) was extracted from healthy young leaves according to [52] followed by RNase treatment (50 µg·mL^−1^). Extracted gDNA was checked for protein, starch or RNA contamination using a Beckmann photospectrometer and ran on a 1% (*w*/*v*) agarose gel in 1 × Tris-acetate-EDTA (TAE) buffer. All DNA probes were labeled with biotin-dUTP or digoxigenin-dUTP by nick translation as described in Mandáková and Lysak [53].

### 2.7. In Situ Hybridization

Selected labelled DNA probes were pooled together, ethanol precipitated, dissolved in a 20 µL mixture containing 50% formamide, 10% dextran sulfate and 2 × SSC, and pipetted onto each of the microscopic slides. The slides were heated at 80 °C for 2 min and incubated at 37 °C overnight. Hybridized probes were visualized through fluorescently-labeled antibodies against biotin-dUTP (red) and digoxigenin-dUTP (green) as in Mandáková and Lysak [53]. Chromosomes were counterstained with 4’,6-diamidino-2-phenylindole (DAPI, 2 µg·mL^−1^) in Vectashield antifade. Fluorescence signals were analyzed and photographed using a Zeiss Axioimager epifluorescence microscope and a CoolCube camera (MetaSystems, Altlussheim, Germany). Individual images were merged and processed using the Photoshop CS software (Adobe Systems). To test the widest possible combination of putative parental species, we performed three series of GISH (*A. alpinum* as one parent + one member of diploids of the *A. aristatum*/*ovatum* complex, the “Mediterranean diploid” and *A. maderense* as the second parent) in several replicates using plants of different provenance.

## 3. Results

### 3.1. Intraspecific Variation in Nuclear Genome Size

**Figure 2:**
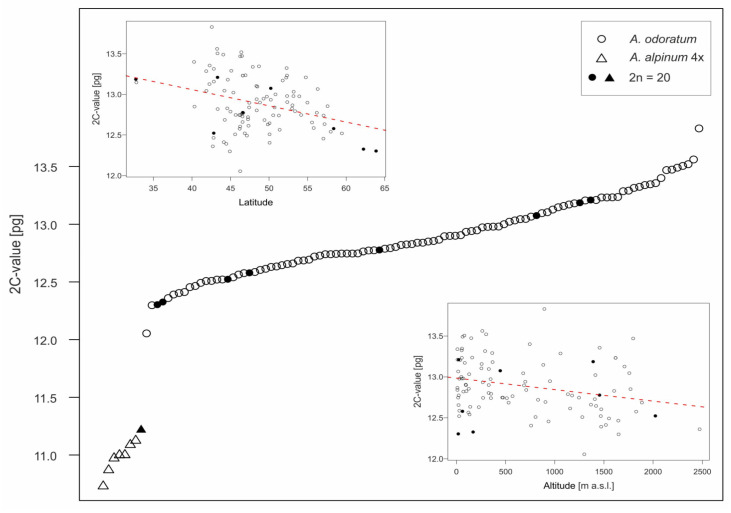
Absolute genome size variation of two tetraploid species studied, *A. odoratum* (open circles) and *A. alpinum* (open triangles). Closed symbols indicate individuals with a counted number of chromosomes. Two insets show the correlation of *A. odoratum* genome size with latitude (top left) and altitude (bottom right).

The genome sizes of both tetraploids formed two non-overlapping groups (Figure 2, Supplementary Table S1), which allowed all individuals to be clearly distinguished, although three mixed populations were found. For *Anthoxanthum odoratum*, 242 plants from 103 populations were analyzed. Mean 2C-values varied from 12.05 to 13.83 pg (mean 12.89 ± 0.34 pg), intraspecific variation in genome size is thus 14.8%. In total, 33 plants from 8 populations were analyzed in *Anthoxanthum alpinum*, mean 2C-values are lower, ranging between 10.73 and 11.22 pg (mean 11.00 ± 0.14 pg DNA) and observed intraspecific variation is 4.6%.

In *A. odoratum*, which was collected from sufficiently large geographic area, the intraspecific variation was non-randomly distributed and showed highly significant negative correlation with latitude and a less pronounced but still significant negative association with altitude (Table 1, Figure 2). Narrow geographic distribution precluded performing the same analyses for 4*x A*. *alpinum*.

### 3.2. Molecular Analyses–Plastid DNA

The lengths of the *trnL-trnF* and the *rpl32-trnL* intergenic spacers in the *Anthoxanthum* full dataset (including 71 specimens plus 3 *Hierochloë* specimens used as outgroups) were 982 and 823 bp, respectively. The combined alignment was 1805 bp long and comprised 129 variable characters including 103 parsimony-informative sites. GenBank accession numbers are provided in Supplementary Table S1.

**Figure 3:**
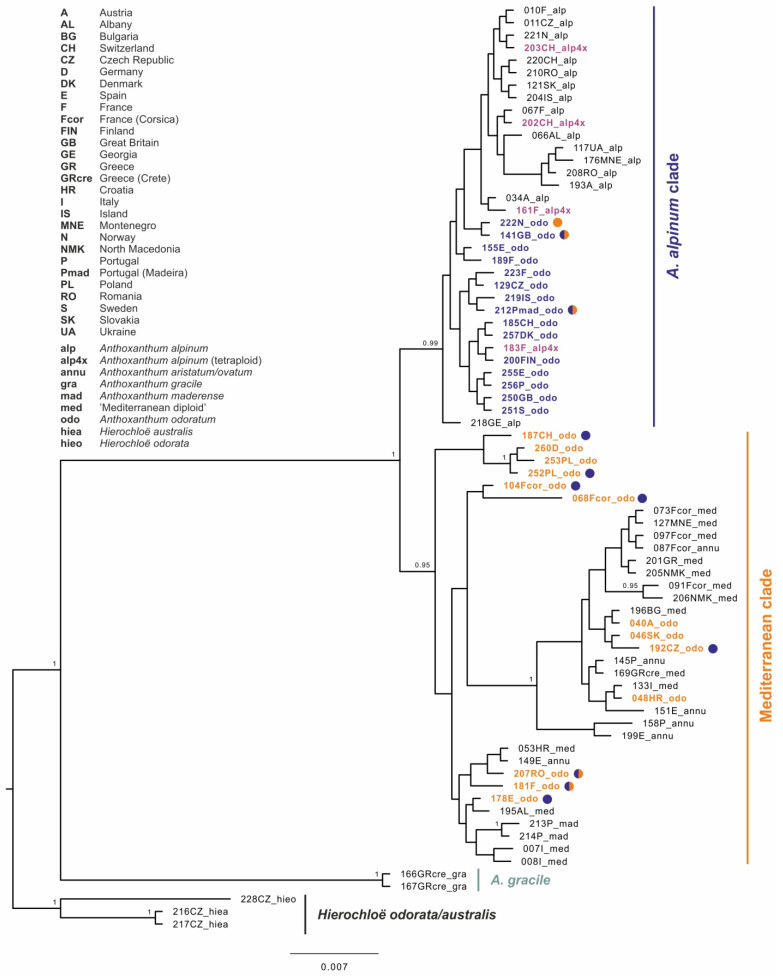
Maximum clade credibility tree obtained from the Bayesian analysis of cpDNA markers (*trnL-trnF* and *rpl32-trnL* intergenic spacers). The numbers by nodes represent Bayesian posterior probabilities if over 0.95. Colored circles indicate individuals carrying a different GBSSI haplotype than the corresponding cpDNA-GBBSI haplotype corresponding to “*A. alpinum* clade” (blue), “Mediterranean clade” (orange) and both clades (half-colored circles).

Hierarchical genetic relationships resolved by Bayesian inference based on the cpDNA dataset shows three main groups of haplotypes within the ingroup. A strongly supported basal group (PP = 1.00) corresponds to the Mediterranean annual diploid species *Anthoxanthum gracile*. The first well-supported bigger group (PP = 0.99, “*A. alpinum* clade”, Figure 3) includes all diploids of *A. alpinum* as well as all tetraploids of *A. alpinum* (labels in bold violet in Figure 3).

It also includes a large proportion of tetraploid *A. odoratum* (labels in bold blue in Figure 3). In sister position to the *A. alpinum* clade, haplotypes of all populations of the “Mediterranean diploid”, annual taxa and *A. maderense* were resolved with the rest of *A. odoratum* (“Mediterranean clade”, PP = 0.95, *A. odoratum* labels in bold orange in Figure 3). Several smaller strongly supported groups can be found in this group compared to the poorly structured *A. alpinum* clade, but do not usually correspond to the different species.

### 3.3. Molecular Analyses–nrDNA–GBSSI

GBSSI alignment in the *Anthoxanthum* full dataset (68 specimens/90 clones + 2 *Hierochloë* as outgroups) was 1166 bp long and comprised 383 variable characters including 268 parsimony-informative sites. In 11 individuals, two different sequence-types were detected and used after cloning analyses. GenBank accession numbers are provided in Supplementary Table S1.

**Figure 4:**
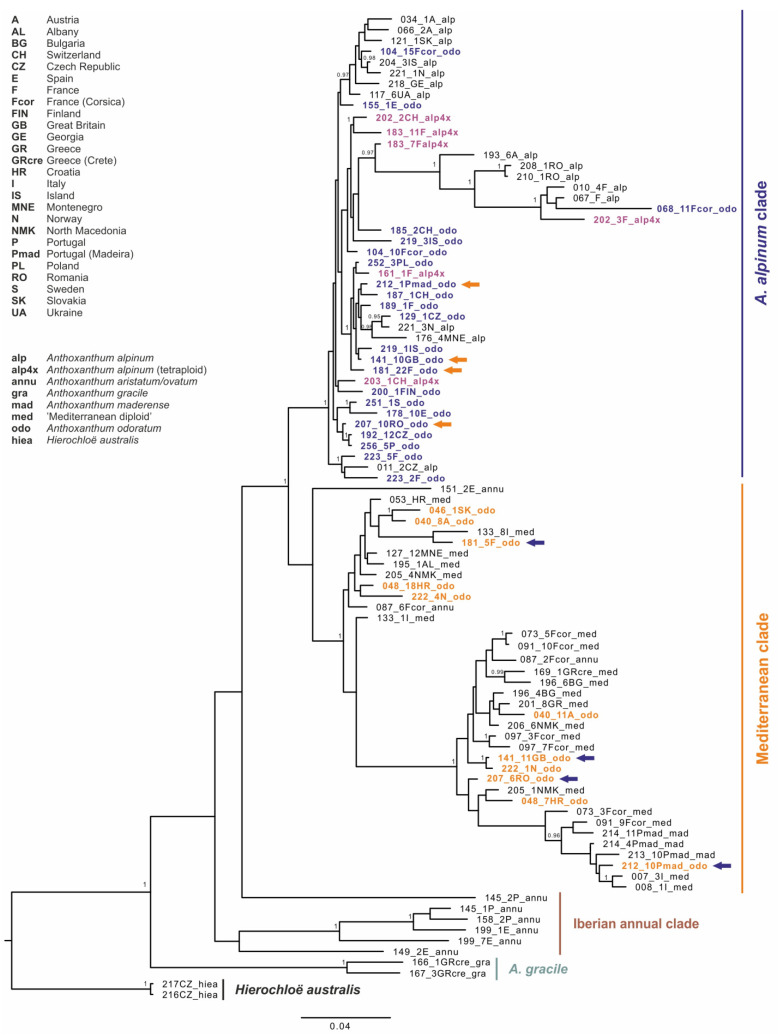
Maximum clade credibility tree obtained from the Bayesian analysis of nuclear DNA marker GBSSI. The numbers by nodes represent Bayesian posterior probabilities if over 0.95. Colored arrows indicate individuals that exhibit two alleles of GBSSI in different clades.

The topology of the Bayesian gene tree based on GBSSI data (Figure 4) revealed three main ingroup haplotype groups with similar pattern to those based on plastid data, but with additional “Iberian annual clade” as a sister group to *A. gracile*. Therefore, we kept similar naming and coloring patterns, and we highlight the differences in GBSSI patterns in *A. odoratum* from those of cpDNA in the Figure 3, where colored circles indicate different positions of the individual within the GBSSI tree. As in the chloroplast tree, *A. gracile* exhibits on the base, all *A. alpinum* plants belong to the “*A. alpinum* clade” and all diploid Mediterranean taxa to the “Mediterranean clade” except for a few annual individuals forming separate lineages. *Anthoxanthum odoratum* splits evenly in both bigger clades and each of the two clones in a different one in some cases (individuals from Great Britain, France, Madeira–Portugal, and Romania; indicated by arrows in Figure 4).

### 3.4. Chromosome Localization of rDNA Loci

**Figure 5:**
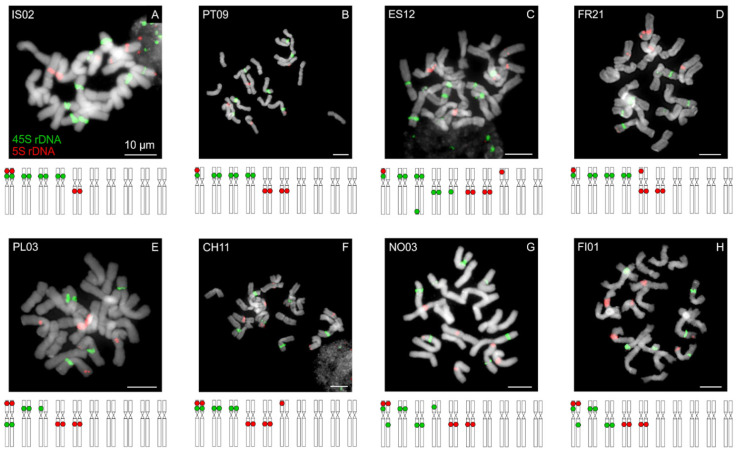
Chromosome localization of rDNA in *Anthoxanthum odoratum*. Mitotic chromosome complements of *A. odoratum* (all 2n = 4*x* = 20) hybridized with 45S (green fluorescence) and 5S (red) rDNA probes. Chromosomes were counterstained with DAPI. Scale bars, 10 µm. Populations from: Island (**A**), Madeira–Portugal (**B**), Spain (**C**), France (**D**), Poland (**E**), Switzerland (**F**), Norway (**G**), Finland (**H**) locality details in Supplementary Table S1.

**Figure 6:**
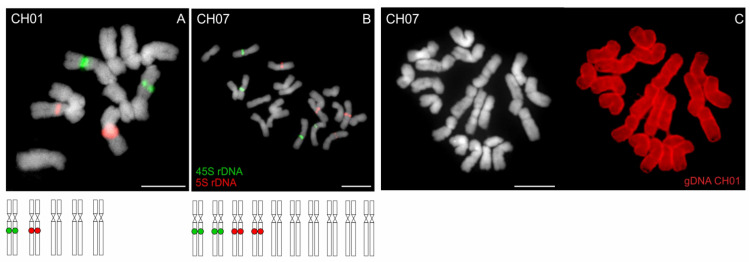
Chromosome localization of rDNA and GISH in *Anthoxanthum alpinum.* (**A**) Diploid population of *A. alpinum* (2n = 10; CH01 from Switzerland) exhibits one 45S- (green fluorescence) and one 5S rDNA-bearing (red fluorescence) chromosome pair. (**B**) Tetraploid population of *A. alpinum* (2n = 20; CH07, Supplemetary Table S1) shows exactly twice the number of rDNA loci compared to the diploid cytotype supporting an autopolyploid origin of the tetraploid. (**C**) GISH in tetraploid *A. alpinum* (CH07) using a probe corresponding to gDNA of diploid *A. alpinum* (CH01, red fluorescence) further suggests an autopolyploid origin of the tetraploid. Chromosomes were counterstained with DAPI. Scale bars, 10 µm.

All of the analyzed *Anthoxanthum* populations have a tetraploid chromosome number (2n = 4*x* = 20; Figure 5 and Figure 6, Figure S1). We uncovered an extensive inter-population variation in the number of 45S (5–9 loci) and 5S rDNA (4–7 loci) and their position on chromosomes (Figure 5, Supplementary Table S1). Geographically more closely related populations show a more similar rDNA pattern, e.g., populations from the Pyrenees and from Madeira (Figure 5B–D), or populations from Norway and Finland (Figure 5G,H) which supports their closer relationship.

Chromosome complement of tetraploid *A. alpinum* contains two pairs of 45S and two pairs of 5S rDNA-bearing chromosomes (Figure 6B). This corresponds exactly to twice the number of rDNA loci compared to the diploid *A. alpinum* cytotype (Figure 6A).

### 3.5. GISH in Polyploid Anthoxanthum Species’

**Figure 7:**
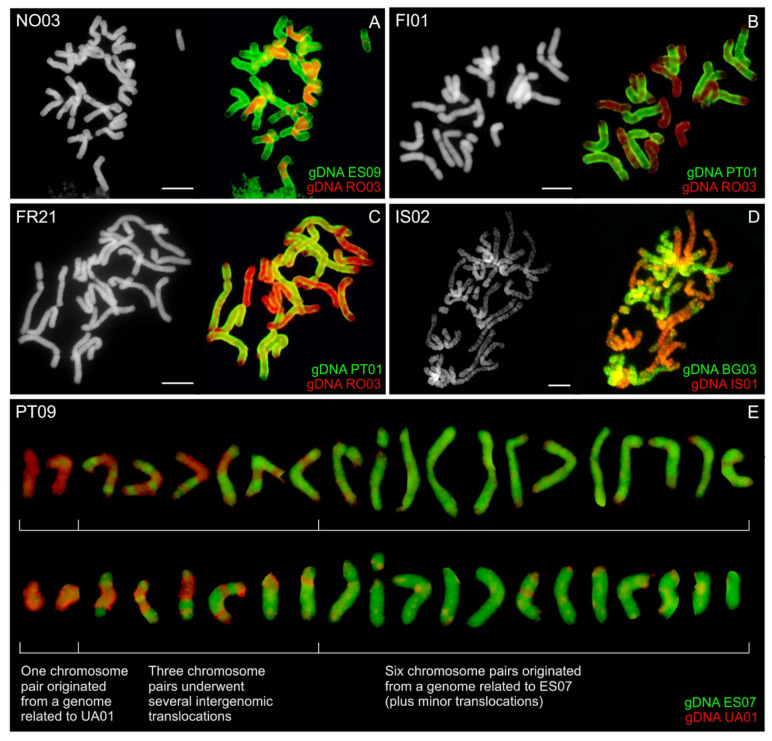
GISH in *Anthoxanthum odoratum.* Mitotic chromosomes of five selected population of *A. odoratum* ((**A**)—NO03, Norway; (**B**)—FI01, Finland; (**C**)—FR21, France; (**D**)—IS02, Iceland; (**E**)—PT09, Madeira–Portugal) were hybridized using gDNA of *A. alpinum* (RO03, IS01 or UA01, red fluorescence in (**A**–**E**) and *A. aristatum/ovatum* (ES09 or ES07; green fluorescence in (**A**,**E**)), *A. maderense* (PT01; green fluorescence in (**B**,**C**)) and “Mediterranean diploid” (BG03, green fluorescence in (**D**)). Chromosomes were counterstained with DAPI. Scale bars, 10 µm.

To uncover the origin of *A. odoratum*, labelled gDNA of all putative diploid parental species was hybridized on mitotic chromosomes of eight tetraploid populations. Following progenitors were tested: diploid *A. alpinum* (RO03, IS01 or UA01), and one of the three Mediterranean diploid species (or its ancestor/s)–“Mediterranean diploid” (BG03, GR04), *A. aristatum*/*ovatum* (ES07, ES09), or *A. maderense* (PT01). The hybridization pattern was highly variable between individual populations of *A. odoratum*, including numerous intergenomic translocations (Figure 7). GISH probe corresponding to *A. alpinum* (red fluorescence in Figure 7 and Figure S1) covered from ¼ (PT09 and Norwegian NO01) up to ½ (in the Icelandic IS02) of the allopolyploid genome, i.e., 5–10 chromosomes were detected using gDNA of *A. alpinum*. The Mediterranean-like genome fraction (green fluorescence in Figure 7 and Figure S1) virtually forms from ½ to ¾ of the hybrid genome (i.e., 10–15 chromosomes) regardless of which of the Mediterranean gDNA probe was used.

In tetraploid *A. alpinum*, GISH using gDNA of diploid *A. alpinum* (CH01 and RO03) hybridized on all 20 chromosomes within the tetraploid chromosome complement, and thus confirmed an autotetraploid origin of *A. alpinum* (Figure 6C, Figure S1).

## 4. Discussion

### 4.1. Intraspecific Variation in Nuclear Genome Size

The issue of intraspecific variation in genome size has been of interest for a long time and still remains somewhat controversial (e.g., [54,55]). In *Anthoxanthum*, intraspecific variation in genome size was observed in all European taxa [3], *A. odoratum* is no exception and with the intraspecific variation 14.8% (Figure 2), it belongs to those species with greater variability.

Several mechanisms are likely responsible for the observed variation in genome size, at least part of the variation can be ascribed to chromosomal heterogeneity, because of presence of aneuploidy and the supernumerary chromosomes was revealed in *Anthoxanthum* in previous studies [3,56]. On the other hand, there is almost no correlation between the number of B-chromosomes and genome size variability in *Anthoxanthum* annuals [56], indicating that the changes mainly in A-chromosomes are responsible for a huge fraction of genome size variability. It is consistent with the fact that we have not found any B-chromosomes during chromosome counting in this study.

The relatively continuous variation in genome size suggested that differences in the size of individual chromosomes have to be involved. Interestingly, the intraspecific variation in genome size in *A. odoratum* is non-randomly distributed and showed highly significant negative correlation with latitude and a less pronounced but still significant negative association with altitude (Figure 2). This finding is highly congruent with the pattern revealed in other diploid *Anthoxanthum* species [3].

Because nuclear genome size can influence several phenotypic and developmental characteristics irrespective of the information coded in the DNA (i.e., the nucleotypic effect; [57]), it can be speculated that the variation found in nuclear genome size represents adaptation to different environmental conditions. Such adaptation may underlie correlations between genome size and abiotic conditions found in several grass genera as *Dactylis* [58,59], *Festuca* [60], *Koeleria* [61], or *Zea* [62]. Obviously, an important source of genome size variability in grasses, i.e., the differential proliferation of the repetitive DNA elements (e.g., [63,64,65]), must also be taken into account. However, data for *Anthoxanthum* are lacking.

### 4.2. Intraspecific Variation in Molecular and Cytogenomic Analyses

Striking intraspecific variation was found in *A. odoratum* in molecular, as well as cytogenomic analyses, which is not surprising due to its polyploid origin. The sequences of *A. odoratum* are highly polymorphic and are present in the clades with *A. alpinum,* as well as with the diploid Mediterranean taxa in the molecular analyses (Figure 3 and Figure 4). In two thirds of cases, individuals exhibit the same pattern within the chloroplast and the nuclear maximum clade credibility tree. However, in the remaining cases, individuals are placed into “*A. alpinum* clade” in the chloroplast tree and into the “Mediterranean clade” in the nuclear tree and *vice versa*, or their GBSSI clones fall into both clades (Figure 3 and Figure 4). In addition, the “Mediterranean clade” is further subdivided into more or less well-supported groups which have been omitted here for simplicity, but the topology depicts relationships among geographically isolated diploid populations [13], and *A. odoratum* can be found in each of them.

In contrast, tetraploid *A. alpinum* shares the pattern in chloroplast and nuclear trees, and in all cases can be found within the “*A. alpinum* clade”.

In terms of cytogenomics, apart from the fact that 20 chromosomes were counted in all samples analyzed, it is difficult to find two sweet vernal grasses with identical rDNA- and GISH-pattern which demonstrates an extensive variation in chromosome structure in *A. odoratum.* Our cytogenetic data suggests that the allotetraploid experienced post-polyploid population-specific intergenomic translocations, probably accompanied by a number of other chromosomal rearrangements that shaped today’s karyotypes. Our findings are in agreement with Jones who pointed out an extreme degree of chromosome differentiation in *A. odoratum* [14]. This study is only a stepping stone for subsequent detailed cytogenetic examination using chromosome-specific markers (e.g., oligopainting).

Although the variability in rDNA location and number among populations remains unresolved, a similar pattern has been observed in some species of the genus *Paspalum* [66] and *Deschampsia* [67,68]. Complexity may be further increased by differences in rDNA variation across the species’ range, which has also been documented in the latter genus ([69] as opposed to [68]).

### 4.3. On the Origin of Anthoxanthum odoratum

Although initially it was thought that *A. odoratum* could be autopolyploid, the opinion slowly began to change and everything pointed to the allopolyploid origin of this taxon, as outlined in the introduction. To confirm the hypothesis of allopolyploid origin, we tried to involve analyses of plastid and nuclear DNA (Figure 3 and Figure 4) that have shown allopolyploid and polytopic origin of *A. odoratum*, as the species exhibits in both main clades (and all subclades in the Mediterranean one) within the trees. Moreover, some of the samples carried at least two alleles of the GBSSI waxy-gene occurring in both (*A. alpinum* and Mediterranean) clades. The observation has similar conclusions to those of Pimentel et al. (2013), with no clear evidence of the parental species. Then we have involved the fluorescence and genomic in situ hybridization (Figure 5 and Figure 7, Figure S1) that have been used in many studies to resolve the origins of polyploids before (e.g., [31]) and in *Anthoxanthum* species by Drapikowska et al. [70]. rDNA pattern (FISH) is highly variable within individual populations of *A. odoratum* (Figure 5) as mentioned above, and only suggests close relationships of some populations. Even if we combine the rDNA pattern of the potential diploid ancestors (diploid *A. alpinum* and the Mediterranean diploid taxa; [13]), we are not able to get the pattern of any *A. odoratum*, ruling out any suggestion of a likely origin.

GISH data (Figure 7) indicate allopolyploid origin and have a highly variable pattern within individual populations, too. On its basis, with a number of visible chromosomal translocations, we can only assume that one of the parental species is A*. alpinum* or its ancestor and the other one is one of the Mediterranean diploids or their common ancestor. The pattern is virtually unchanged on each individual of *A. odoratum* using genomic probes from the three species mentioned, so we can just take into consideration the annual life cycle of *A. aristatum/ovatum* and geographical distance of *A. maderense* to suggest that the most likely parent is the “Mediterranean diploid”. The pattern points to various scenarios of the tetraploid origin ranging from equal to noticeably unbalanced contribution of both parents. In two thirds of accessions analyzed it theoretically seems to possible fusion of a reduced gamete of *A. alpinum*-like genome (bearing five chromosomes) with an unreduced gamete of “Mediterranean diploid”-like genome (bearing 15 chromosomes; Figure 7E). On the other hand, the possible long existence of tetraploids may have contributed to the fact that the current GISH pattern does not correspond to the state at their origin, but rather to their later evolution.

Current distributional ranges of putative parental taxa points to hybridization events in the past that could begin by divergence of *A. alpinum* (2.02 Mya; [13]). Interestingly, similar timing of polyploidization events was shown by Tusime et al. [11] in the African polyploid *Anthoxanthum nivale*.

### 4.4. Autopolyploid Origin of Anthoxanthum alpinum

The autopolyploid origin of tetraploid *A. alpinum* was first suggested on the basis of the analyses of its karyotype by Teppner [26] and by Hedberg [24]. In addition, investigations on the flowering phenology [32] and on the sensitivity to a specific rust showed greater similarity in the two cytotypes of *A. alpinum*, compared to related species “Mediterranean diploid” and *A. odoratum* [33], thus corroborating the autopolyploid origin of *A. alpinum*. The close genetic similarity testing in 1999 by Zeroual-Humbert-Droz and Felber [34] using isozymes between the diploid and tetraploid cytotype confirmed the autopolyploid origin of the tetraploid, too. The study of Chumová et al. [3] provides additional support for this hypothesis by revealing almost identical mean monoploid genome sizes of diploid (1Cx = 2.76 pg) and tetraploid (1Cx = 2.75 pg) cytotypes of *A*. *alpinum*. The position within the “*A. alpinum* clade” on chloroplast and nuclear tree (Figure 3 and Figure 4), as well as the pattern shown in Figure 6 as the result of fluorescence and genomic in situ hybridization on diploid and tetraploid *A. alpinum* from Switzerland, also supports this hypothesis.

### 4.5. Consequences of Allopolyploidy in A. odoratum

Despite long-lasting debate on the polyploid origin of *A. odoratum*, our study represents the first rigorous evidence on allotetraploidy in its evolutionary history. We have shown the contribution of *A. alpinum* (or its most recent common ancestor) as one parental species and the participation of a second, as yet ambiguous parental diploid species from the Mediterranean. The gathered evidence points very likely to so-called “Mediterranean diploid” (this study and [3,13]) that was repeatedly recorded in the past as diploid *A. odoratum* [16,24,32,33]. This name can no longer be used, because we have definitively rejected the hypothesis of an autotetraploid origin of *A. odoratum*. The species name “*odoratum*” is restricted to the allotetraploid taxon only. Unlike *A. alpinum*, where the species name “*alpinum*” can be used for both (diploid and tetraploid) cytotypes due to its autopolyploid origin. In line with our previous studies [3,13], we call for a thorough and detailed description of the “Mediterranean diploid” and its proper recognition for science as a new species. Its taxonomic value is well supported by vicariant species range compared to tetraploid *A. odoratum* and other diploid species [13], by perennial life-cycle [14] and the genome size [3].

## 5. Conclusions

By combining flow cytometry, molecular analyses and fluorescence and genomic in situ hybridization, we pointed out the large intraspecific variability in *Anthoxanthum odoratum* and were able to confirm its allopolyploid origin. We also provide further evidence for the autopolyploid origin of tetraploid. *A. alpinum*. We have suggested the possible parental species of both tetraploids, noting that in some cases these are rather the ancestors of existing species, so their exact identification is probably impossible.

## Figures and Tables

**Table 1:** Pearson’s correlation with corresponding *p*-values (significant values in bold) for population distribution data (latitude, longitude, and altitude) and mean population genome sizes (2C-values) for *Anthoxanthum odoratum*.

Population Characteristics	t-Value (df = 101)	*p*-Value	Correlation Coefficient
Latitude	−3.42	**<0.001**	−0.322
Longitude	−0.69	0.505	−0.066
Altitude [m a.s.l.]	−2.80	**0.006**	−0.269

## Data Availability

The data presented in this study (DNA sequences) are provided in GenBank under accession numbers MZ399726-MZ399797 and MZ408552-MZ408642 (details in Supplementary Table S1).

## Supplementary Materials

The following are available online at https://www.mdpi.com/article/10.3390/genes12070966/s1, Table S1: Overview of *Anthoxanthum odoratum* populations used in the study. In addition to the georeferenced data, the results of the genome size estimation and the codes used in each analysis are added for each population. Moreover, the table includes information on diploid accessions from previous studies used for phylogenetic analyses; Figure S1: The GISH pattern of particular individuals of *A. odoratum* and one tetraploid *A. alpinum* (bottom right pane) obtained by hybridization of gDNA from different combinations of putative parental species. Individuals from each population of *A. odoratum* are in rows, while the three different parental combinations are arranged in columns.
