# Supplementary figures and images for: On the Origin of Tetraploid Vernal Grasses (Anthoxanthum) in Europe
Source: Genes (Basel). 2021 Jun 24;12(7):966. doi: 10.3390/genes12070966 (PMC8308110; doi:10.3390/genes12070966)

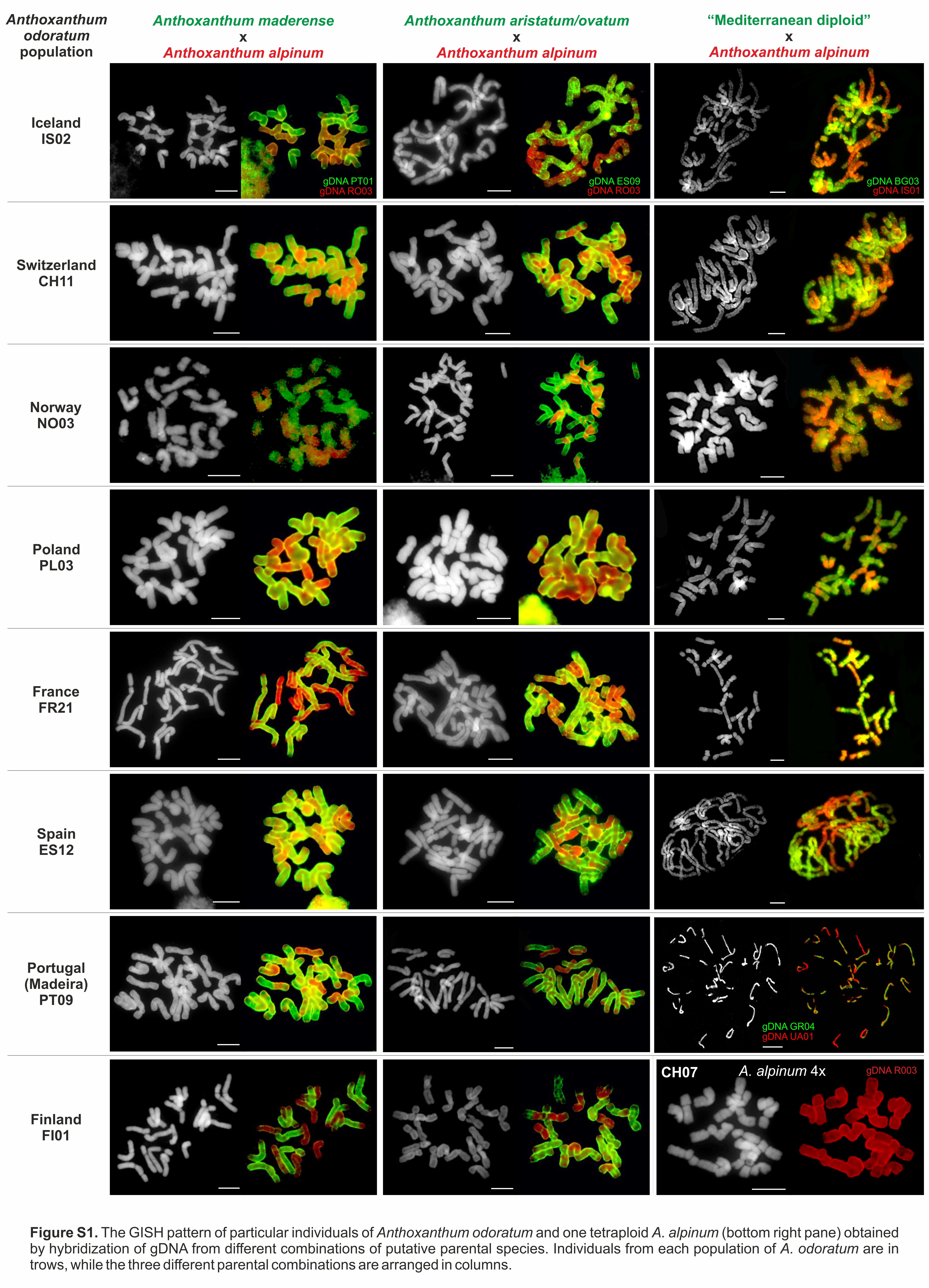

Supplement: Supplementary file 1 [file genes-12-00966-s001.zip › Supplementary_material/Figure_S1.jpg]
